# Pulse Wave Analysis Method of Cardiovascular Parameters Extraction for Health Monitoring

**DOI:** 10.3390/ijerph20032597

**Published:** 2023-01-31

**Authors:** Ji Jin, Xingguang Geng, Yitao Zhang, Haiying Zhang, Tianchun Ye

**Affiliations:** 1 The Institute of Microelectronics, Chinese Academy of Sciences, Beijing 100029, China; 2 University of Chinese Academy of Sciences, Beijing 100049, China

**Keywords:** pulse wave analysis, multiple cardiovascular parameters, health monitoring, waveform recognition, improved DTW

## Abstract

Objective: A pulse waveform is regarded as an information carrier of the cardiovascular system, which contains multiple interactive cardiovascular parameters reflecting physio-pathological states of bodies. Hence, multiple parameter analysis is increasingly meaningful to date but still cannot be easily achieved one by one due to the complex mapping between waveforms. This paper describes a new analysis method based on waveform recognition aimed for extracting multiple cardiovascular parameters to monitor public health. The objective of this new method is to deduce multiple cardiovascular parameters for a target pulse waveform based on waveform recognition to a most similar reference waveform in a given database or pattern library. Methods: The first part of the methodology includes building the sub-pattern libraries and training classifier. This provides a trained classifier and the sub-pattern library with reference pulse waveforms and known parameters. The second part is waveform analysis. The target waveform will be classified and output a state category being used to select the corresponding sub-pattern library with the same state. This will reduce subsequent recognition scope and computation costs. The mainstay of this new analysis method is improved dynamic time warping (DTW). This improved DTW and K-Nearest Neighbors (KNN) were applied to recognize the most similar waveform in the pattern library. Hence, cardiovascular parameters can be assigned accordingly from the most similar waveform in the pattern library. Results: Four hundred and thirty eight (438) randomly selected pulse waveforms were tested to verify the effectiveness of this method. The results show that the classification accuracy is 96.35%. Using statistical analysis to compare the target sample waveforms and the recognized reference ones from within the pattern library, most correlation coefficients are beyond 0.99. Each set of cardiovascular parameters was assessed using the Bland-Altman plot. The extracted cardiovascular parameters are in strong agreement with the original verifying the effectiveness of this new approach. Conclusion: This new method using waveform recognition shows promising results that can directly extract multiple cardiovascular parameters from waveforms with high accuracy. This new approach is efficient and effective and is very promising for future continuous monitoring of cardiovascular health.

## 1. Introduction

The cardiovascular system has complex biomechanical relationships, which can affect the physio-pathological states of publics. The pulse waveform is regarded as the information carrier of the cardiovascular system, and it is affected by the internal coupling between the heart and vascular [1,2,3]. Moreover, during the propagation in the arterial circulation, arterial stiffness and wave reflections at arterial junctions can also influence the blood flow distribution and change the pulse waveform [4]. Pulse wave analysis can extract effective cardiovascular information from the pulse waveform, revealing cardiovascular function and health conditions.

To date, many methods have been developed for pulse wave analysis for extracting cardiovascular information, including blood pressure assessed directly on the waveform with some initiate calibration. A variety of analysis methods are derived based on the basic concepts of wave reflection [5] or the arterial Windkessel model [6], which provide some parameters such as augmentation index (AIx) and vascular compliance to describe the cardiovascular system [7,8,9,10,11]. The computation involved is more dependent on the location accuracy of feature points. Other analysis methods include extracting a series of time-domain or frequency-domain features from the pulse waveform, and then these waveforms are briefly divided into several categories by machine learning which represents some specific body conditions [12,13]. There are also some methods for direct estimation of single cardiovascular parameters such as cardiac output (CO), which require calibration using other auxiliary equipment or empirical coefficient [14,15,16,17]. The above analysis methods have advantages and disadvantages in terms of the difficulty in the method implementation complexity and the analysis accuracy. 

The pulse waveform is a complex physiological signal in the human circulation system. The waveform is influenced by the interaction of left ventricular stroke volume, systemic vascular resistance, vascular compliance, and other cardiovascular system characteristics [18], which comprises a complex relationship among them [19]. In general, the characteristic above can be demonstrated by various cardiovascular parameters. When one single characteristic changes, it often leads to associative changes in others [20]. It becomes difficult to analyze the effect of waveforms from a single characteristic. It is also difficult to analyze a specific correlation between these cardiovascular parameters. For example, AIx is widely used to measure wave reflection, and the reflected wave is largely determined by pulse wave velocity (PWV). However, they are not simply interchangeable parameters of arterial stiffness, because AIx is also influenced by vasoactive drugs independently of PWV. Automated simultaneous measurement of these parameters are useful for risk stratification of hypertensives [21]. The usual way of extracting multiple parameters from waveforms is to calculate the individual parameters separately. Some studies have tried to build the independent mapping relationship between the features of waveforms and a single parameter, but it shows many limitations. For some parameters, in order to achieve good agreement between extracted and actual value, repeated calibrations using additional equipment to compensate the effects by other cardiovascular characteristics are required. While other studies are more dependent on the location accuracy of feature points, which is more susceptible by noise and by the diversity of waveforms. Hence it is difficult to extract these cardiovascular parameters accurately just based on the features of only parts of waveforms. Multiple cardiovascular parameters extraction when fully developed, can provide more information of the complex interacted cardiovascular system. Hence, there is excellent potential for an automated multiple cardiovascular parameters extraction method since the overall process can be efficient, effective and hence cost and time saving. However, there are associated limitations and challenges to overcome.

In this paper, a new pulse wave analysis method for monitoring public health is proposed. This approach will directly extract multiple cardiovascular parameters from the radial artery pulse waveform. This method uses the whole time series of the waveform containing more detailed information, not only some certain specific partial feature points. The key idea of this method is to recognize the most similar reference waveform in pattern library with the target waveform and then assign cardiovascular parameters accordingly. The methodology can be divided into two parts: (i) preparation work and (ii) waveform analysis. The preparation work is essentially building the sub-pattern libraries which stores a large number of reference pulse waveforms with known cardiovascular parameters (including, cardiac output, carotid-radial PWV, augmentation index (AIx), augmented pressure (AP), reflection index (RI) and stiffness index (SI)) under the same state and training a classifier with machine learning method of LibSVM. It can narrow down the recognition range for the next part. The mainstay of this new method is the improved DTW. The waveform analysis introduced an improved DTW and KNN to seek out the most similar reference pulse waveform in the selected sub-pattern library that contains a series of cardiovascular parameters for assigning to the target pulse waveform. 

## 2. Methods

**Figure 1:**
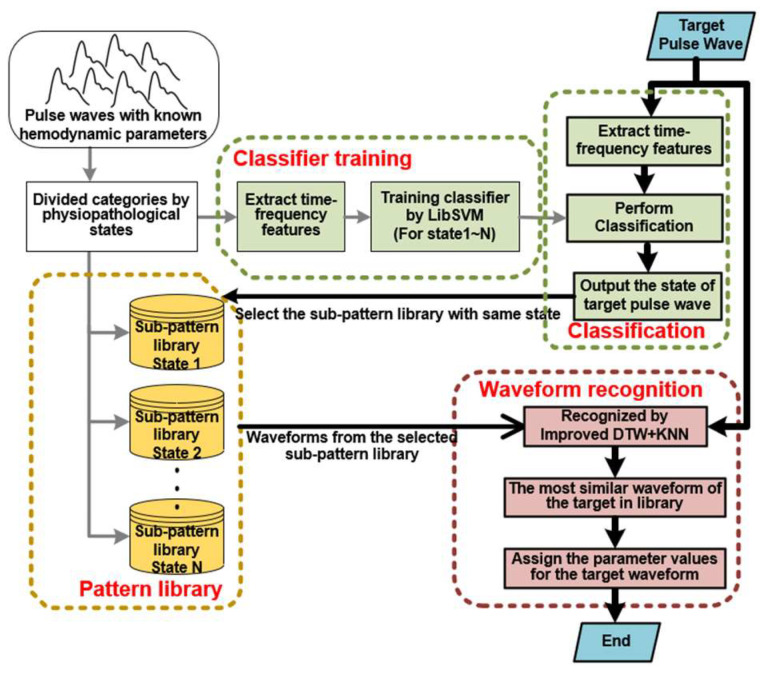
Flowchart methodology.

This method can be mainly divided into two parts: (i) preparation work and (ii) waveform analysis. The part (i) includes building sub-pattern libraries and a training classifier. The part (ii) contains the classification and waveform recognition. The detailed flowchart of this method is shown in Figure 1. 

At first, the preparation work includes building sub-pattern libraries and a training classifier. A large number of reference pulse waveforms with known cardiovascular parameters are divided into different categories according to physio-pathological states. The reference waveforms of each state will be stored in the sub-pattern library. At the same time, the time-frequency features of the waveform are extracted, which are used for the machine learning LibSVM to train a classifier model. This model is used to automatically classify the categories when the state of target pulse waveform is unknown in the next part of waveform analysis. 

In the waveform analysis, the time-frequency features of target waveform are extracted and put into the training classifier. After classification, a state category label will be output and used to select the corresponding sub-pattern library with same state. It can narrow the searching scope for the next step of waveform recognition and improve the algorithm efficiency. The waveform recognition uses an improved DTW and KNN to recognize the most similar reference waveform from the selected sub-pattern library. Lastly, once the most similar pulse waveform is recognized and selected, respective cardiovascular parameters can be assigned accordingly.

### 2.1. Preparation Work

The preparation work is time consuming. However, when completed it will be used directly for every target waveform in the next part. It contains the sub-pattern library and training classifier. It saves the reference pulse waveforms with known parameters as sub-pattern library directly through the different category of states, which is similar to a waveform dictionary. At the same time, it extracts the features with wavelet transform method, and uses the different categories of states as labels. Then, these features and state labels will be input into the machine learning method of LibSVM to train the classifier. The detail of feature extraction and classifier training are shown below. 

#### 2.1.1. Time-Frequency Feature Extraction with Wavelet Transform

**Figure 2:**
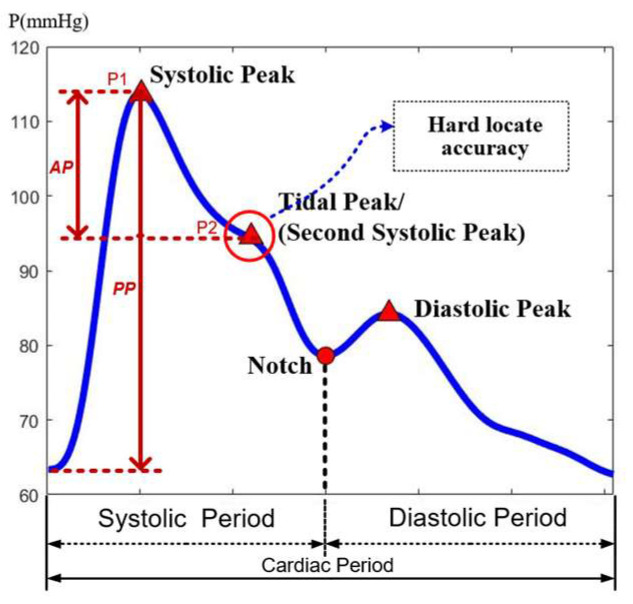
Pulse waveform and location of feature points.

The pulse waveform mainly includes two peaks and a notch, between the first peak and notch having a tide peak, which often exists as an inflection point and occasionally juts out as a peak point, as shown in Figure 2. Most feature extraction methods in the time-domain often locate these points and calculate their relative positions as feature parameters [22]. The accurate locating of the tide peak is very important in calculating parameters. However, since the location of feature points varies greatly from waveform to waveform, it is not uncommon to obtain erroneous results due to incorrect location. Another feature extraction method in the frequency-domain can extract the main frequency components of the pulse waveform, but it would ignore the abrupt change points of the signal in the time domain. 

Wavelet transform is a multi-scale method that can display the frequency components in different time periods. The low-frequency interval is represented on a small scale, and the high-frequency interval is represented on a large scale, which can simultaneously meet the needs of time-frequency analysis.

**Figure 3:**
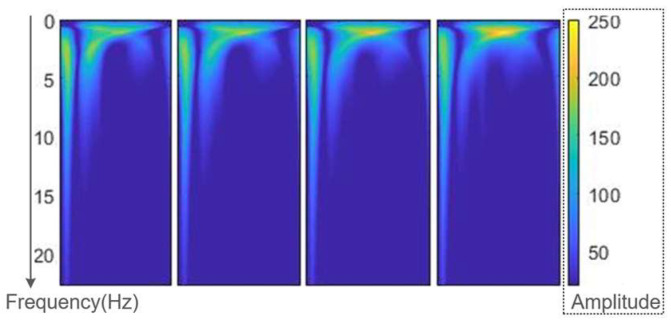
Time-frequency diagrams of four pulse waveforms.

The frequency of pulse waveform is mainly distributed at the range of 0~20 Hz, and 99% spectral energy of signals is concentrated in the range of 0~10 Hz [23]. The time-frequency plot of the continuous wavelet transform can show the variation of the frequency components with time. Figure 3 shows four different pulse waveforms. It can be seen that the differences in the frequency component between these waveforms are also concentrated in the above-mentioned range. Therefore, the main components will be extracted from this frequency range by decomposing the pulse waveform with a discrete wavelet transform. 

**Figure 4:**
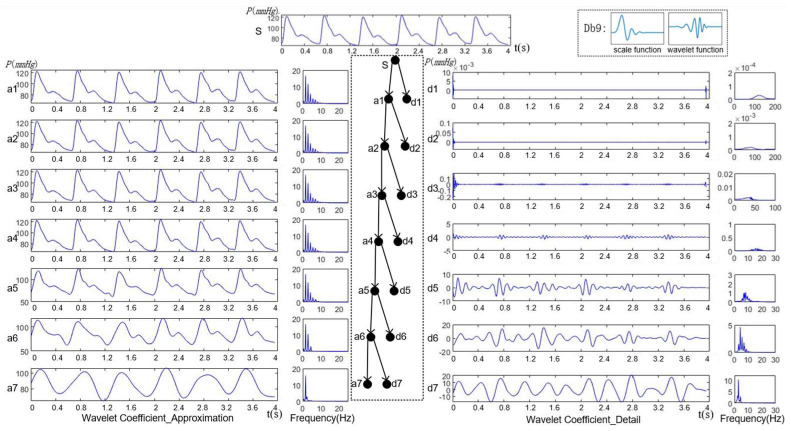
Wavelet decomposition of pulse waveform.

It selects the Db9 wavelet as the wavelet base, which is similar in shape to the pulse waveform [24]. The pulse waveform is decomposed into seven layers by this wavelet base as shown in Figure 4. The layers above seven are generally considered to be the baseline drift caused by low-frequency noise that would be eliminated in the signal preprocessing. The a1~a7 represents the low-frequency segment in each layer called the approximation of signals. The d1~d7 represents the high-frequency segment in each layer called the detail of signals. The original signal S can be viewed as being decomposed into (d1~d7) + a7. From the frequency components of each layer, it can be seen that the waveform is basically close to the original form of pulse waveforms since the fourth layer a4 = (d5~d7) + a7. It has included the main frequency components within 0–10 Hz. Moreover, in the same layer, the high-frequency component of d4 is about 10–20 Hz, whose amplitude is less than 1% of the original signal. It shows that the components d5~d7 and a7 can be used to represent most of the pulse waveform information. So, the wavelet coefficients of these components contains the main features of pulse waveforms.

The pulse waveform is decomposed into different frequency intervals. It can hierarchically display the waveform characteristics of each frequency interval in the time domain. The low-frequency components contain the basic change trend, and the high-frequency components contain detailed information. The time-series signal is converted to the time-frequency domain, so that the different frequency segments of signal are presented in the wavelet coefficients arranged in time order, which can be used as the time-frequency features for state classification.

#### 2.1.2. Training Classifier with LibSVM

Support Vector Machine (SVM) is a supervised classification technique based on optimal margins in machine learning. The goal of SVM is to find a hyperplane with the largest margin between the two-class boundary line, enabling binary classification. For the features of non-linear inseparable samples, it is hard to find a hyperplane to classify [25]. So, this kind of feature needs to be mapped to a high-dimensional space to become linearly separable and then be classified in high dimensions. In the high-dimensional space, the sample distance can be directly calculated through a kernel function without explicitly displaying the mapping function. The kernel function can return the result of the inner product of the mapped samples, as shown in the Equation (1).

(1) $$K\left(x1,x2\right)=\phi {\left(x1\right)}^{T}\phi \left(x2\right)$$

where *ϕ*(·) is the mapping function, K represents the kernel function, and *x*1 and *x*2 are the two data. 

**Figure 5:**
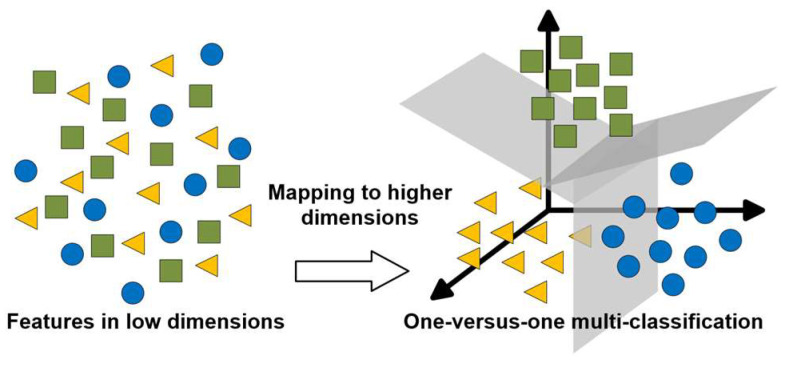
Multi-classification diagram.

In many practical problems, not only binary classification is required, but also multi-classification. It needs to divide the samples into multiple categories. LibSVM is a software library for SVM classification, which can be loaded into several software platforms such as Matlab [26]. It can directly implement multi-classification using one-versus-one (OVO) technology based on binary classification. Figure 5 shows the diagram of multi-classification. The left side represents features that are non-linearly inseparable in low dimensions, and the right side is the high-dimensional feature space after mapping, which is easier to find hyperplanes to separate features into multiple classes.

In the process of training, the label of each pulse waveform with different physio-pathological states categories is added. Next, the extracted time-frequency features are combined with these labels as a training set for the classifier. They are input to the LibSVM to build a set of hyperplanes as the model of classifier. The determination of the state category of test waveform basing on the position of its time-frequency feature relative to this set of hyperplanes. After training, a classifier capable of directly classifying multiple classes will be formed. It can be used to automatically classify the category of pulse waveforms with unknown state in the pulse wave analysis. 

### 2.2. Waveform Analysis

After preparation work, the trained classifier and stored sub-pattern library will be used for each target waveform. This part of waveform analysis contains the classification and waveform recognition. Through classification, the state category of target waveform can be obtained. It will be used to select the corresponding sub-pattern library with the same state. Next, carry out pulse waveform recognition for every reference waveform in this sub-pattern library with target waveform one by one to recognize the most similar one. Lastly, to assign the values of corresponding cardiovascular parameter of the most similar one for the target pulse waveform. Therefore, the step of classification can narrow down the scope of recognition. It just needs to perform the recognition process for only one sub-pattern library with the same state. The details of waveform recognition are described below.

#### Waveform Recognition with Improved Dynamic Time Warping and KNN

The whole time series contains more useful information, which may be lost in the process of extracting features. Therefore, it is necessary to use the pulse waveforms with whole time series for recognition so as to find most similar reference one in pattern library. 

Dynamic time warping (DTW) is a non-linear distance measure method that maps one series onto another to find the best alignment path between two series by warping the time dimension [27]. This method is very robust to the changing in the time axis and amplitude axis, which is very suitable for pulse wave signals with different frequencies and similar timing waveforms. It can calculate the difference between the target waveform and each reference waveform in the sub-pattern library. The mainstay of this study is an improved DTW method that focus more on the pulse waveform morphological difference, which can be converted to a value of distance between two pulse waveforms. Following, the morphological difference combines with heart rate and pulse pressure as input features for the KNN to find the most similar reference one to the target pulse waveform. 

**Figure 6:**
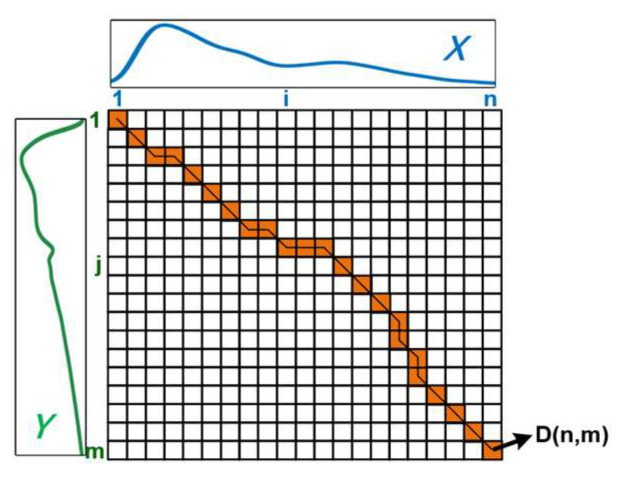
Dynamic time warping.

The implementation process of DTW is to build a distance matrix for two time series. Figure 6 shows the form of the DTW distance matrix D. Two pulse waveforms of length *n* and *m* are represented by time series *X* = *x*1, *x*2, …, *xn* and *Y* = *y*1, *y*2, … *ym*. Measure the distance dis(*xi*, *yj*) between each point *xi* of *X* and the point *yj* of *Y*, and then accumulate the minimum distance of previous points to fill the distance matrix *D*. The matrix size is *n* × *m*. Each element in the matrix *D* is the cumulative distance, and the calculation method can be seen in the following two equations: (2) and (3).

*D*[*i*,*j*]= *dis*(*xi*,*yj*) + *min*(*D*[*i* − 1,*j*],*D*[*i*,*j* − 1],*D*[*i* − 1,*j* − 1]) (2)

*Distance* = *Sum*(*shortest pathsXY*) = *D*[*n*,*m*] (3)

The last element of *D*[*n*,*m*] is the value of the shortest distance. After calculating all the elements in the matrix *D*, returns from *D*[*n*,*m*] to D[1,1] to find the path of shortest distance between these two series. The red path in Figure 6 displays the shortest path.

**Figure 7:**
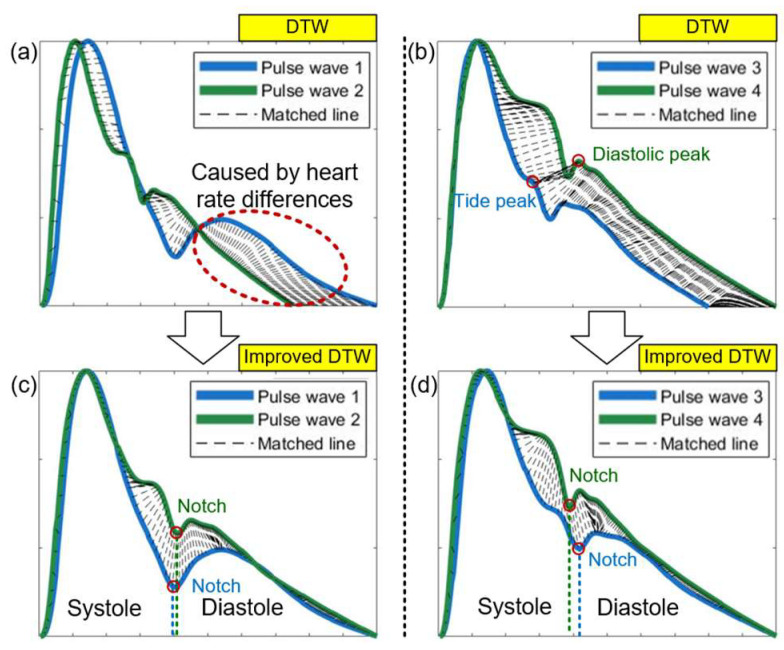
Matched points between two pulse waveforms using DTW (**a**,**b**) and improved DTW (**c**,**d**).

When directly using DTW to calculate the distance between two pulse waveforms, the connection of matched points for the shortest path is shown in Figure 6. The cumulative distance between two pulse waveforms is greatly affected by heart rate differences. At the end-diastolic period of waveform, the actual difference between these two waveforms in the tail is small, but their different heart rates lead to the tail being the main part of cumulative distances. It is much larger than the actual morphological difference, as shown in Figure 7a. Therefore, the cumulative distance will be affected by heart rate, which could severely reduce the proportion of morphological difference in the cumulative distances, affecting the waveform recognition result and also the pulse pressure.

The matched points would be connected in different periods when the two pulse waveforms have large morphological differences. For example, as shown in Figure 7b, the shortest path is mapped from the tide peak in the systole of pulse wave 3 to the diastole peak of pulse wave 4. This would cause the mapping path not correlating well with the actual physical meaning contained in waveforms. In order to solve the above problems, the DTW method needs to be improved.

To focus more on analyzing the morphological difference and avoid the interference of heart rate and pulse pressure, both the amplitude and time axis are normalized before DTW is carried out.

In the pulse waveform, there is a notch that marks the boundary between systole and diastole, which is a very obvious feature point. The generation of this point is caused by the activity of cardiovascular system. When the heart valve closes, the pressure at the aortic root drops sharply, then rises back up, creating a distinct notch. It propagates through the arterial tree to become this notch. In order to compare pulse waveforms at the same period, this point should be marked as *x*(*i*) and *y*(*j*) on the time axis of the two pulse waveforms, respectively. In the distance matrix *D*, it is the element of *D*[*i*,*j*]. Then, it constrains the shortest path that must pass through this point, so that the notch point of one waveform will be mapped to another notch point. The two sides of this point are systolic period and diastolic period representing the ejection process and after the blood ejection stops respectively. This boundary point isolates the two periods avoiding the cross-mapping at different periods. After applying the improved DTW, changes in the pulse waveform can be seen as follows: from Figure 7a to Figure 7c with improved DTW, and from Figure 7b to Figure 7d with improved DTW. The computation of distances, after applying the improved DTW becomes more consistent with the actual morphological differences.

With the improved DTW method, the cumulative distance between the target pulse waveform and each reference waveform in the selected sub-pattern library is calculated, which represents the waveform difference between two pulse waveforms. Moreover, the improved DTW method focuses more on the morphology differences. However, in the process of normalizing amplitude and time of the waveform, heart rate and pulse pressure information are removed. Therefore, they would be used as independent features combined with the morphological differences feature into a feature group. Then, use the KNN to recognize the most similar one to the target pulse waveform.

KNN can find some data close to the target data’s features. These data are called nearest neighbors, and k is the number of selected nearest neighbors [28]. When the KNN is used for waveform recognition, the selection of the k-value means how many waveforms need to be selected that close to the target pulse waveform. If it needs to recognize the most similar waveform, k is set to 1 directly.

**Figure 8:**
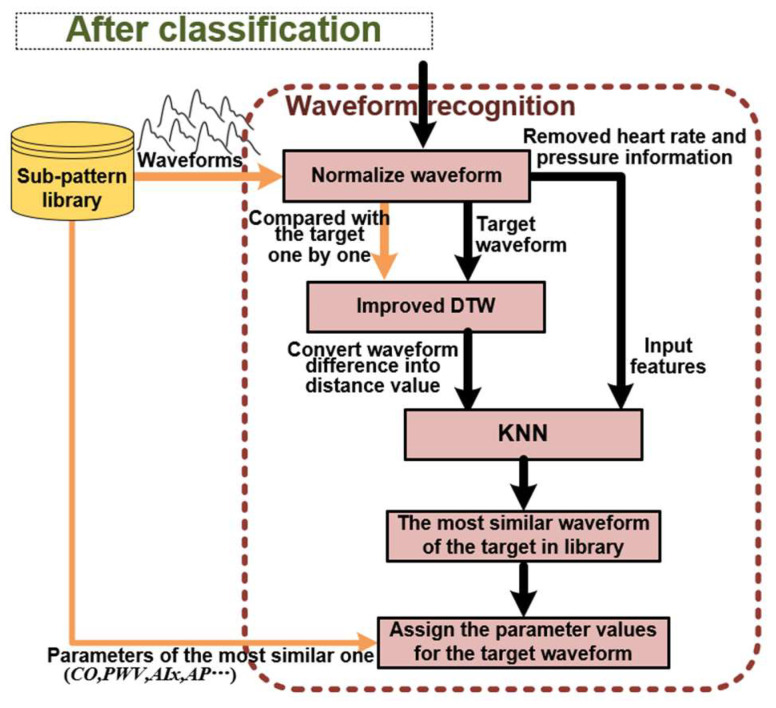
Waveform recognition process.

In this method, DTW is used to convert the waveform difference into the distance, and KNN utilizes it in the feature space to search for the nearest data. The combination of these two methods has natural advantages. Moreover, basing on the DTW has been improved, it can focus more on the morphological difference according to the actual physiological meaning of pulse waveforms. It also combines the removed information of heart rate and pulse pressure as other features to search for the most similar pulse waveform through KNN. Lastly, cardiovascular parameters can be extracted from similar reference waveforms with the same state. Assign the parameter values of the most similar one for the target waveform. After this final step, multiple cardiovascular parameters can be extracted based on waveform recognition. Detail of the implementation process is shown in Figure 8.

Both the DTW and KNN require one-to-one analysis between each waveform stored in the pattern library and the target waveform. This process consumes high computational time. In actual situation, the number of pulse waveforms determines the computation cost. Therefore, in this new method, classification and sub-pattern library selection can reduce the recognition scope and decrease the calculation cost significantly.

## 3. Case Results

In this section, examples of pulse waveforms with different physiological status will be tested to verify the effectiveness of this newly developed method.

Aging has a direct influence on both arterial distensibility and compliance which can be inferred by arterial stiffness, and arterial stiffness is an established parameter responsible for cardiovascular disease. An increase in arterial stiffness will lead to an increase in arterial pressure and heart load. This will alter the cardiovascular parameters considered in this study. Hence, the pulse waveforms through selected randomly will be grouped according to age.

The newly developed method will be applied to the randomly selected pulse waveforms, then comparing the extracted results with the original cardiovascular parameters.

### 3.1. Data and Statistics

In this work, the set of data comprises 4374 virtual healthy subjects pulse waveform aged from 25 to 75, with corresponding cardiovascular parameters [8]. Of which, 3936 data are stored in the sub-pattern library under six different age categories as reference waveforms. These are used as training classifier. The remaining 438 pulse waveforms are tested with the new method using waveform recognition.

All steps of this method and the statistical analysis of results are completed by the software of MATLAB version 2018b (The MathWorks Inc., Natick, MA, USA). The classification results use the listed confusion matrix to calculate the accuracy rate. In the results of waveform recognition, the similarity of waveforms is checked visually and make statistics by Pearson correlation analysis. The agreement between the original parameters and the parameters extracted by this method are displayed by the Bland-Altman plot. These results of the example are used to test the accuracy of the pulse wave analysis method for extracting cardiovascular parameters.

### 3.2. Classification

In the classifier training, the optimal values for setting classifier parameters are obtained through grid optimization, which is beneficial in preventing the model of classifier from overfitting or underfitting. The performance of classification is tested by age states of these randomly selects 438 sample waveforms. After the classification by the trained classifier, compare the classification results with the actual age states.

The confusion matrix of classification results is shown in Table 1. The labels of 1~6 represent the six different age states, which is divided into six groups from the age 25 to 75. After calculation, the accuracy rate is 96.347%. The recall, precision, and comprehensive evaluation index F1-score are all greater than 96%, as shown in below Equations (4)–(7), which indicate a good performance of the classifier.

(4) $$accuracy=\frac{1}{6}\sum_{i=1}^{6}\frac{TP_{i}+TN_{i}}{TP_{i}+TN_{i}+FP_{i}+FN_{i}}=96.347\%$$

(5) $$recall=\frac{1}{6}\sum_{i=1}^{6}\frac{TP_{i}}{TP_{i}+FN_{i}}=96.29\%$$

(6) $$precision=\frac{1}{6}\sum_{i=1}^{6}\frac{TP_{i}}{TP_{i}+FP_{i}}=96.35\%$$

(7) $$F_{1}\_Score=\frac{1}{6}\sum_{i=1}^{6}2\frac{recall_{i}*precision_{i}}{recall_{i}+precision_{i}}=96.31\%$$

where *TP_i_* is the true positives for *i*th class, which is the number of predictions that data labelled to belong to the *i*th class was correctly classified as this class. *TN_i_* is the true negatives, which is the number of predictions that the data labelled non-*i*th is correctly classified as a non-*i*th class. *FN_i_* is the false negatives, which is the number of predictions that the data labelled *i*th is incorrectly classified as a non-*i*th class. *FP_i_* is the false positives, which is the number of predictions that the data labelled non-*i*th class is incorrectly classified as the *i*th class.

### 3.3. Waveform Recognition

Before the process of recognition, the pulse waveform will be normalized. For the time axis of waveforms, it uses the down sampling to make every waveform with the same number of points. According to the Nyquist-Shannon sampling theorem, the sampling rate should be more than twice the frequency range of the signal. In actual applications, the sampling rate should be more than five times to ensure retaining the fully biomedicine signal information. The frequency of pulse waveforms is mainly distributed at 0~20 Hz. So, the sampling rate should be maintained at least 110 Hz after down sampling. The normal heart rate is 60~100 bpm and one pulse waveform has the time range of 0.6~1 s. In some situations, the time range is going to be larger. To make sure the sampling rate is not less than 110 Hz after down sampling, the number of points within each waveform at least 150. Hence, the least sampling rate of the original signal should be more than 250 Hz. In this example, the sampling rate of pulse waveform is 500 Hz, which is enough for this method. So, in the normalizing process, all of these waveforms are down sampled to 200 points. After the normalization, the removed information of heart rate and the pulse pressure will be input to the KNN as features later.

**Figure 9:**
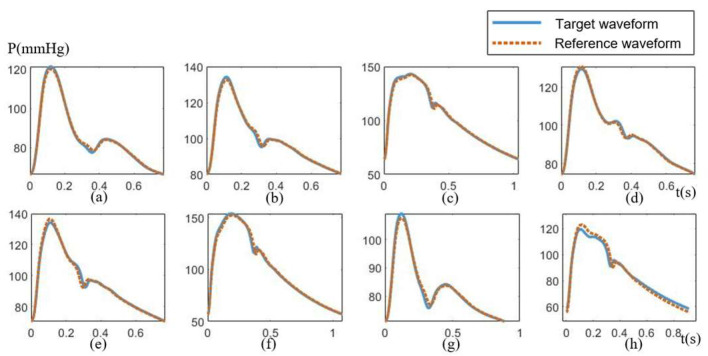
Reference waveforms match well with targeted waveforms. (**a**–**h**) 8 groups selected randomly comparing the recognized reference waveforms and matching well with the target waveforms.

For each of the 438 sample test pulse waveforms, the most similar reference pulse waveform will be sought and recognized from the sub-pattern library. The results showed that sample test pulse waveform is similar to the recognized waveforms from the library. This is broadly illustrated in Figure 9 with 8 groups selected randomly comparing the recognized reference waveforms and matching well with the target waveforms.

**Figure 10:**
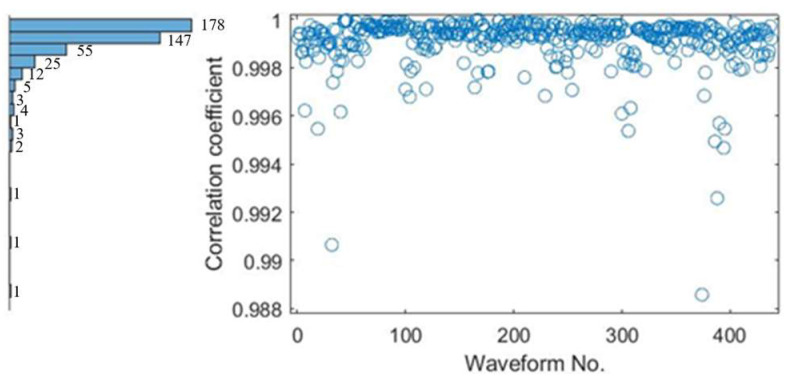
The distribution of correlation coefficients of 438 groups waveforms.

The 438 groups of waveforms were analyzed by Pearson correlation analysis to show their similarity and statistics. The statistical results of the correlation coefficient are shown in the Figure 10 and Table 2 (all *p* < 0.005, paired *t*-test). All of them have a very high correlation between the target waveforms and the recognized reference one.

The cardiovascular parameters include Cardiac output (CO), Carotid-Radial PWV, augmentation index (AIx), augmented pressure (AP), reflection Index (RI) and stiffness index (SI) were used to test the accuracy of this pulse wave analysis method for extracting cardiovascular parameters. Lastly, the extracted parameters were compared with the original parameters of target waveforms.

**Figure 11:**
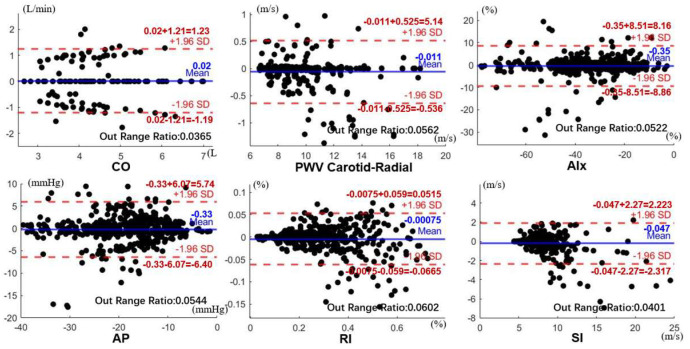
The Bland-Altman plot of six cardiovascular parameters.

The Bland-Altman plot is a simple and intuitive graphical method for displaying the data agreement of extracted parameters and real original parameters. In the Bland-Altman plot, the vertical axis is the difference between the two sets of data, and the horizontal axis is the average value. The blue solid line is the mean differences. When the blue line is closer to the 0 value, it means that the two sets of data have a better agreement. The upper and lower red horizontal dashed lines are the bounds of ±1.96 standard deviations of the mean difference, which is also the 95% confidence interval of the mean difference. It is used to determine the precision of results. Figure 11 shows the agreement analyze results of these six cardiovascular parameters, respectively. For each parameter, 438 sets of data are within the narrower limits of confidence interval suggesting that they are reasonably comparable. The number of points out of the ±1.96 standard deviations range is near the ratio of 0.05. This verifies that the data almost fit the normal distribution with about 95% data in the confidence interval. The mean value of the differences around 0 indicates that the mean differences of parameters between the extracted and the original are negligible. The results of Bland-Altman plots show the two sets of cardiovascular parameters in agreement with each other.

### 3.4. Comparison with Conventional Method

A comparison between this newly method and conventional method was carried out. AIx and AP were selected as the test cardiovascular parameters for comparison.

The conventional method essentially computes the peak points pressure [29]. The peak points are located by the way of finding the zero-crossing point of derivative waveforms or finding the max curvature points of waveforms. The amplitude of two peak points P1 and P2 are used to calculate parameters of AIx and AP, as shown in the Figure 2. For an accurate calculation, the peak feature points in each waveform have been adjusted manually to make sure every point was located in the correct position.

AP = P2 − P1(8)

AIx = AP/PP × 100%(9)

**Figure 12:**
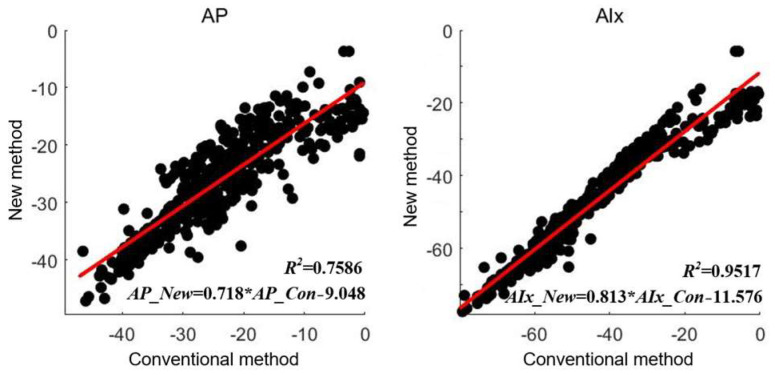
Relationship between two methods.

The results of parameters AP and AIx of 438 data are extracted by these two methods. The comparison was carried out using the Pearson correlation analysis as shown Figure 12. The horizontal axis represents the calculated parameters value by conventional method and the vertical axis are the extracted parameters value by this new method. The Pearson correlation coefficients between the two results are 0.8710 and 0.9755 of AP and AIx (all *p* < 0.001, paired *t*-test). The correlation between the two method is good for Augmented Pressure (AP) and excellent for Augmentation Index (AIx).

For the conventional method, the calculation of these two parameters relies on the accuracy of peak points. However, the tide peak is always an inflection point which can be easily affected by noise in the location process. This position can have large variation from waveform to waveform. If use one location way applied to this peak point, it can work for some waveforms, but other types of pulse waveform, the location of the peak will most likely be in the wrong location. There will always be a need to adjust the peak location manually. Hence, using the conventional method to locate this point will require several iterations.

This newly developed method using waveform recognition can be automated conveniently to extract cardiovascular parameters from a reference waveform in a pattern library. This approach saves both time and computational cost.

## 4. Discussion

The improved DTW that focus more on the pulse waveform morphological difference is applied to pulse waveform recognition. Basing on pulse waveform recognition, it does not need to locate the tide peak points. Pulse recognition approach can be automated and hence the methodology can be applied to handle large scale data. Another advantage is that it does not need additional auxiliary equipment to calibrate.

A comparison between this new method and conventional method [29] yielded strong correlation hence verifying the effectiveness of using the pulse waveform recognition technique.

The drawback of this pulse waveform recognition method is that the pulse waveform library needs to have voluminous pulse waveforms with known cardiovascular parameters. If this can be achieved, then this novel method holds great potential as the overall process can be automated hence effective and efficient in deriving cardiovascular parameter from pulse waveform.

A natural extension of this study is to include a large number of pulse waveforms of different cardiovascular diseases with known corresponding cardiovascular parameters. According to the main disease types, these waveforms with known parameters will be saved as sub-pattern libraries and used to train the classifier to output the disease category for the unknown waveform. Then, the most similar waveform from the corresponding sub-pattern library will be recognizable with the same state to assign the parameters for the unknown waveform of new subject. It can be used for analyzing public cardiovascular health with clinical outcomes at different pathological states to find new possibilities for cardiovascular disease prediction and health monitoring.

## 5. Conclusions

In this study, a new pulse wave analysis method is proposed, applying waveform recognition method, to seek the most similar reference pulse waveform from the pattern library, and assign the cardiovascular parameters for the target pulse waveform accordingly.

Waveform recognition using an improved DTW is the mainstay of this project. The improved DTW method focused more on the morphology difference of pulse waves. It also combines KNN and using pulse pressure and heart rate as independent features to increase recognition accuracy to find the most similar waveforms. This approach greatly enhanced the outcome of the results.

The effectiveness of this method was verified by 438 randomly selected pulse waveforms. The effectiveness of the classifier tested by comparing the predicted states and the actual data to reach accuracy rate greater than 96%. In addition, each set of the cardiovascular parameters is assessed using the Bland-Altman plot and almost all showed strong agreement. The results show strong correlation between extracted cardiovascular parameters from recognized reference pulse waveform and the actual data. This outcome results verify the effectiveness of the method. However, more rigorous tests will be carried out and the method will also be expanded to include diseased pulse waveforms.

Using pulse waveform recognition approach to extract cardiovascular parameters can be efficient and effective, hence saving time and cost during operation. It can be used for public daily health and clinical continuous monitoring.

## Figures and Tables

**Table 1:** Confusion matrix of classification.

Confusion Matrix		Predict Class					
		1	2	3	4	5	6
Actual Class	**1**	64	3	-	-	-	-
	**2**	2	60	2	-	-	-
	**3**	-	1	72	2	-	-
	**4**	-	-	3	77	-	-
	**5**	-	-	-	3	76	-
	**6**	-	-	-	-	-	73

**Table 2:** The correlation statistics result of 438 groups of waveforms.

Correlation Coefficient	1–0.998	0.998–0.996	0.996–0.994	0.994–0.992	0.992–0.988
number	405	24	6	1	2

## Data Availability

Not Applicable.
